# Chronic Functional Adaptations Induced by the Application of Transcranial Direct Current Stimulation Combined with Exercise Programs: A Systematic Review of Randomized Controlled Trials

**DOI:** 10.3390/jcm12216724

**Published:** 2023-10-24

**Authors:** Daniel Marcos-Frutos, Virginia López-Alonso, Irene Mera-González, José Andrés Sánchez-Molina, David Colomer-Poveda, Gonzalo Márquez

**Affiliations:** Department of Physical Education and Sport, Faculty of Sports Sciences and Physical Education, University of A Coruña, 15179 A Coruña, Spain; danielmarcosfrutos@gmail.com (D.M.-F.); virginia.lopez.alonso@udc.es (V.L.-A.); irene.mera@udc.es (I.M.-G.); jose.andres.sanchez.molina@udc.es (J.A.S.-M.)

**Keywords:** noninvasive brain stimulation, time-to-task failure, maximal voluntary contraction, primary motor cortex, prefrontal cortex

## Abstract

The present systematic review aimed to determine the chronic effects of the combination of transcranial direct current stimulation (tDCS) and exercise on motor function and performance outcomes. We performed a systematic literature review in the databases MEDLINE and Web of Science. Only randomized control trials that measured the chronic effect of combining exercise (comprising gross motor tasks) with tDCS during at least five sessions and measured any type of motor function or performance outcome were included. A total of 22 interventions met the inclusion criteria. Only outcomes related to motor function or performance were collected. Studies were divided into three groups: (a) healthy population (n = 4), (b) neurological disorder population (n = 14), and (c) musculoskeletal disorder population (n = 4). The studies exhibited considerable variability in terms of tDCS protocols, exercise programs, and outcome measures. Chronic use of tDCS in combination with strength training does not enhance motor function in healthy adults. In neurological disorders, the results suggest no additive effect if the exercise program includes the movements pretending to be improved (i.e., tested). However, although evidence is scarce, tDCS may enhance exercise-induced adaptations in musculoskeletal conditions characterized by pain as a limiting factor of motor function.

## 1. Introduction

Noninvasive brain-stimulation techniques have gained attention in neuroscience and clinical research due to their ability to modulate cortical excitability and influence various cognitive and motor functions. Among these techniques, transcranial direct current stimulation (tDCS) involves the application of low-intensity (1–3 mA) constant electrical currents to specific cortical areas through two electrodes on the scalp [1,2]. This safe, painless, and noninvasive technique [3] can induce modulations in cortical excitability lasting up to 90 min with just a 13 min application [4,5]. Furthermore, tDCS polarity, whether anodal (a-tDCS) or cathodal (c-tDCS), can, respectively, increase or decrease the resting membrane potential of the targeted brain area [1].

The versatility of tDCS has sparked research interest across various fields, such as cognitive enhancement [6] or pain management [7]. However, a special focus has been directed towards investigating its immediate (i.e., acute) effects on motor function, including rehabilitation [8] or sports performance [9,10,11,12,13,14,15]. For example, among the healthy population, several systematic reviews and meta-analyses suggest that a single session of tDCS may increase performance in several motor tasks, like endurance time to exhaustion, visuomotor skills, and strength [9,10,11]. Other populations, like adults with neurological disorders, may also benefit from the acute effects of tDCS. For example, Beretta et al. [7] revealed moderate improvements in postural control and balance following a single session of tDCS in adults with neurological disorders.

However, most of the research conducted to date has primarily focused on the acute effects of a single tDCS session [12,13,14,15]. These acute effects may be especially relevant in contexts where immediate performance is crucial, such as competitive sports. Nevertheless, tDCS could be systematically employed to induce or enhance the chronic adaptations derived from other interventions like exercise. The acute effects of tDCS may increase the quality of gross motor-task performance (depending on the task) during training sessions, thus optimizing or accelerating motor-skill acquisition or exercise-induced adaptations [12]. This may be relevant not only in the field of sports performance but also for populations like adults with neurological or musculoskeletal disorders, in which initial motor performance deficits may compromise the quality of life and/or rehabilitation.

However, evidence about the potential benefits of systematically (i.e., chronically) incorporating tDCS into exercise protocols is scarce and controversial. Two studies did not report any effect of tDCS on motor performance after 12 sessions of tDCS combined with exercise in stroke survivors [16,17]. However, Wang et al.’s [18] systematic review revealed that tDCS led to greater improvements in the dynamic postural stability index following a period of 4–6 sessions of postural training and stimulation in healthy subjects. Additionally, the variability in experimental designs, combined with the lack of long-term evidence, poses challenges in synthesizing studies for meta-analyses [19,20].

Therefore, the aim of the present systematic review is to determine the chronic effects of combining tDCS with exercise (comprising gross motor tasks) on motor function and performance outcomes. We hypothesize that the immediate effects of tDCS before or during exercise, when used systematically during a training or rehabilitation period, would chronically enhance motor function and performance to a greater extent than exercise alone. We addressed this aim through a systematic literature search that identified three main different populations where the effects on motor function of chronic tDCS in combination with exercise have been investigated: (a) healthy population, (b) neurological disorder population, and (c) musculoskeletal disorder population.

## 2. Methods

This systematic review adheres to the Preferred Reporting Items for Systematic Reviews and Meta-Analyses (PRISMA) statement [21].

### 2.1. Search Strategy

A systematic literature search was conducted on the US National Library of Medicine (PubMed) and Web of Science databases up to 28 March 2023. The search strategy employed the following terms: (exercise OR “resistance training” OR endurance OR aerobic OR “strength training” OR running OR cycling) AND (a-tDCS OR anodal-tDCS OR c-tDCS OR cathodal-tDCS OR tDCS OR “transcranial direct current stimulation”). The search was performed by D. M.-F. In cases of uncertainty, a second author (G. M.) was involved in the process until a consensus was reached. The authors of the selected articles were contacted to request any relevant missing information.

### 2.2. Eligibility Criteria and Study Selection

After the removal of duplicates, the titles and abstracts of the remaining articles were screened. Subsequently, the full texts of the obtained reports were evaluated. The following inclusion criteria had to be met in the studies: (a) published in English, (b) adult population (aged over 18), (c) randomized control trials, (d) the intervention protocol combined exercise with tDCS, (e) the protocol mainly comprised gross motor tasks (e.g., gait, dumbbell biceps curl, or cycling), (f) measurement of any type of motor function or performance outcome before and after the intervention (e.g., grip strength, knee extension peak torque, or 10 m walk test time), and (g) at least 5 sessions conducted during the intervention. Studies involving cognitive interventions were excluded.

### 2.3. Data Collection and Extraction

After study selection, the essential characteristics of the trials were reported in three tables, including the main author and year of publication, sample (size, age, and gender), tDCS protocol, exercise protocol, and outcomes. Only outcomes related to motor function or performance were collected. If any study had more than two intervention groups, only data from the sham stimulation plus exercise group and the real stimulation plus exercise group were collected. The participants’ inclusion criteria were reported in the tables for neurological and musculoskeletal disorder populations.

For significant results, the corresponding *p*-value was recorded. Whenever possible, the level of significance from the magnitude of change comparison or effect x time interaction was extracted to compare the tDCS effect with the sham group.

### 2.4. Risk of Bias and Quality of Evidence Assessment

The risk of bias and the methodological quality of the included studies were evaluated using the Physiotherapy Evidence Database (PEDro) scale [22]. Studies with a score of ≥6/10 were considered “high quality”, while those with lower scores were categorized as “low quality”. The methodological quality of each study was assessed by D. M.-F. In case of uncertainty, a second author (G. M.) participated in the rating process until a consensus was reached.

## 3. Results

### 3.1. Search Results

**Figure 1:**
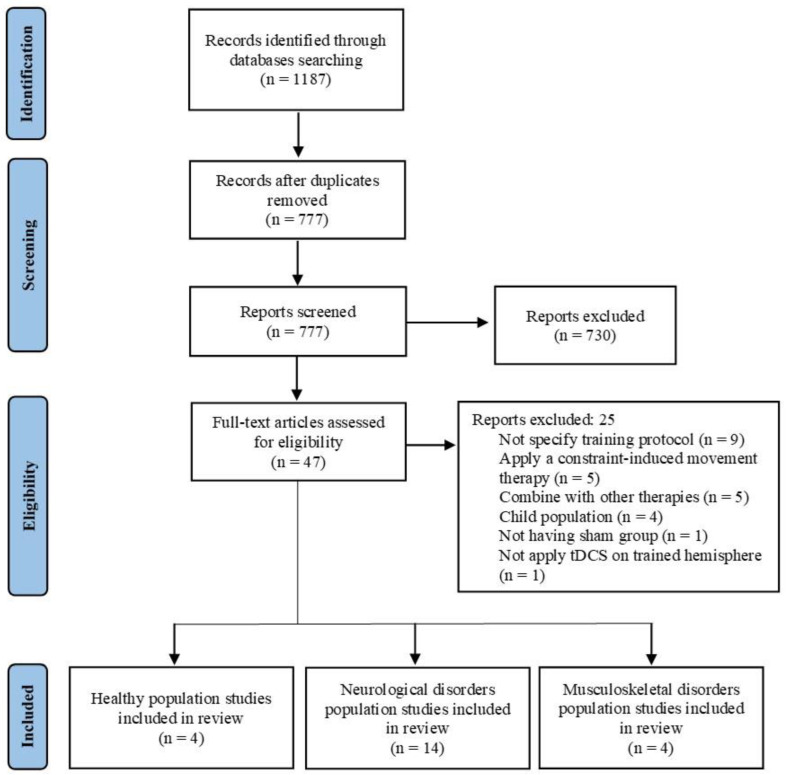
Flow diagram of the studies that underwent the review process.

Figure 1 shows the flow diagrams for the entire search process. Initially, 1187 studies were identified (575 in PubMed and 612 in Web of Science). After removing duplicates, 777 studies remained. A screening of titles and abstracts resulted in 47 studies for full-text screening. Ultimately, 22 studies that met the inclusion criteria were selected. After performing a qualitative analysis, studies were divided into three groups based on population characteristics: (a) healthy population (n = 4), (b) neurological disorder population (n = 14), and (c) musculoskeletal disorder population (n = 4).

### 3.2. Risk of Bias and Methodological Quality of Studies

All the included studies obtained a PEDro score between 6 and 9 points, indicating a “high methodological quality” (mean score: 7.59 ± 0.91). The most frequently omitted items were the “intention to treat” analysis (16 studies), blinding of therapists (12 studies), blinding of assessors (7 studies), and allocation was concealed (6 studies) (Table 1). Notably, several studies were double blinded without specifying the second blind (i.e., therapist or assessor). In such cases, it was assumed that the assessors were blinded and the therapists were not.

### 3.3. Healthy Population

#### 3.3.1. Participants and Study Characteristics

Table 2 presents the participants and study characteristics of the healthy population. The total number of participants was 129 (49 M, 50 W, and 30 unspecified [26]). The mean age of the subjects ranged from 20 to 26 years, except for the study by Jung et al. [19], where the mean age ranged from 39 to 40 years.

Most studies applied conventional a-tDCS or high-definition tDCS (HD-tDCS) [26]. The target electrode was placed over the primary motor area (M1) for a-tDCS (intensity: 2 mA; surface electrode: 25–28 cm^2^) or at Cz for HD-tDCS (intensity: 2 mA; surface electrode: 3.14 cm^2^). All studies, except one [24], applied tDCS online.

The training protocols varied significantly across the studies (session range: 7–21). The study by Jung et al. [19] implemented a “strength-endurance” circuit training. The other studies involved different strengthening exercises, such as dumbbell wrist extension at 70% 1 RM [23], knee flexion and extension with a maximum intention on an isokinetic machine at 30°/s [25], or various foot-core exercises [26].

#### 3.3.2. Primary Outcomes

No study reported greater improvements in strength [23,24,25,26], “strength-endurance”, Sargent jump height [24], or balance [26] in the real tDCS group compared to the sham group. Only Xiao et al. [21] reported greater improvement in toe flexor strength (31 ± 19% vs. 9 ± 17%) (Table 2).

### 3.4. Neurological Disorder Population

#### 3.4.1. Participants and Study Characteristics

Table 3 summarizes the participants and study characteristics of the neurological disorder population. The total number of participants was 409 (237 M, 210 W) with a mean age ranging between 40 and 73 years. Among the participants, 258 were stroke patients, 109 had multiple sclerosis, 22 were Parkinson’s disease patients, and 20 were old people with mild cognitive impairment.

The tDCS was applied over M1 (intensity: 1–2.5 mA; surface electrode: 12.5–35 cm^2^) [16,17,27,29,30,31,33,35,36,38], 3 cm lateral to the inion (intensity: 2 mA; surface electrode: 25–35 cm^2^) [32,34], supplementary motor area (intensity: 1 mA; surface electrode: 25 cm^2^) [28], or 2 cm anterior to the vertex (intensity: 2 mA; surface electrode: 35 cm^2^] [37]. Wong et al. [26] applied a-tDCS on ipsilesional M1 or c-tDCS on contralesional M1 in stroke patients. Five studies applied online stimulation, while nine studies were offline (see Table 3).

The exercise programs varied across the studies (session range 6–36). The main components of the protocols involved gait [27,28,31,34,37], strength training alone [16,17,30] or combined with treadmill walking [29,33], circuit training combined with treadmill walking [32], cycling [36], elliptical ergometer [35], or Tai Chi [38].

#### 3.4.2. Primary Outcomes

The combination of tDCS with exercise in patients with neurological disorders shows varying effects across different outcome measures. In stroke patients, no significant additional effects were observed in dexterity [16,17], spasticity [16], range of motion [17], or balance [27]. However, tDCS showed additional effects over just exercise in the Fugl-Meyer Assessment in three studies [29,30,31], but not in the other four [16,17,27,28]. Similarly, tDCS plus exercise had no additional benefits for the Wolf Motor-Function Test in one study [16] but led to greater improvements in the functional ability score in another one [30]. tDCS did not add further benefits for the Trunk Impairment Scale and Performance-Oriented Mobility Assessment than just exercise [28].

Regarding strength in stroke patients, tDCS enhanced the effect of exercise on knee flexion and extension peak torque of the paretic limb but not on the nonparetic limb, further reducing extension but not the flexion bilateral deficit [29]. However, grip strength [16,17,30], the strength of different joint actions, and performance in the Five Times Sit-to-Stand Test [30] did not benefit from adding tDCS to exercise.

Adding tDCS to exercise improved the gait speed of stroke patients in two studies [28,29] but not in the other three [27,30,31]. Similarly, the Timed Up-and-Go Test performance benefited from adding tDCS to exercise in one study [28] but not in two other studies [27,30]. No additive effects were found when gait speed was measured while patients performed other motor tasks (i.e., motor dual-task gait) [31].

Cardiovascular fitness, measured as external work performed at maximum oxygen consumption or at the gas-exchange threshold, showed greater improvements in the tDCS group in one study [29]. However, there were no significant differences in maximum oxygen consumption or oxygen consumption at the gas-exchange threshold in the same study.

In multiple sclerosis patients, balance improved more in the tDCS group in one study [33]. Regarding functional tests, the Timed Up-and-Go Test improved more in the tDCS group in one study [36] and did not differ in two studies [32,34], and there was no comparison between groups in one study [33]. The Figure-of-Eight Walk Test and Dynamic Gait Index did not differ between groups in one study [32]. tDCS had a significant effect on gait speed in one study [35] and did not have an effect in another study [34], and the significance differed on the method of assessment in two studies (i.e., gait speed vs. distance covered in a 10 m Walk Test and a 2 min Walk Test vs. 5 m Walk Test, respectively) [35,36].

In Parkinson’s disease patients, the only study included did not report significant improvements in bradykinesia, Timed Up-and-Go Test, gait speed, or balance in the real tDCS group compared to the sham group [37].

In old people with mild cognitive impairment, the only study included did not report significant improvements in normal gait speed or in motor dual-task walk speed [38].

### 3.5. Musculoskeletal Disorder Population

#### 3.5.1. Participants and Study Characteristics

Table 4 summarizes the participants and study characteristics of the musculoskeletal disorder population. The total number of participants was 112 (32 M, 80 W). The mean age of the participants ranged from 20 to 25 years, except for the study by Chang et al. [36], where the age range was 60–64 years. Among the participants, 54 had chronic ankle instability, 30 had knee osteoarthritis, and 28 were women experiencing patellofemoral pain.

Most studies applied conventional a-tDCS or HD-tDCS [40]. The target electrode was allocated to M1 (intensity: 1–2 mA; surface electrode: 15–35 cm^2^) [39,41] or Cz (intensity: 2 mA; surface electrode: 0.79 cm^2^) [40,42]. Two studies employed online tDCS, while the other two offline tDCS (see Table 4). All studies implemented a strength-training program (session range 10–16).

#### 3.5.2. Primary Outcomes

In individuals with chronic ankle instability, balance improved more in the tDCS group in one study [40], but not in the other one [39]. However, there were no significant differences between groups in ankle proprioception [40], strength, and Side Hop Test [39].

For individuals with knee osteoarthritis, the only included study reported a greater improvement in the tDCS group for the Western Ontario and McMaster Universities Osteoarthritis Index Physical Function Subscale [41].

In the only study that included women with patellofemoral pain, the tDCS group exhibited a significant improvement in strength compared to the sham group [42].

## 4. Discussion

The purpose of this systematic review was to examine the chronic effects of combining tDCS with exercise on motor function and performance. We hypothesized that systematic application of tDCS before or during exercise over a training or rehabilitation period would chronically enhance motor function and performance to a greater extent than just exercise alone. Our findings suggest that combining tDCS with exercise, compared to just exercise, (a) did not demonstrate greater chronic effects on performance in healthy individuals; (b) the effects on function and performance in neurological disorder populations varied depending on the task tested, but overall results suggest modest or null additive effects when exercise is specific enough to the motor function that wants to be improved; and (c) enhanced the effects of exercise over function in musculoskeletal conditions characterized by pain as a limiting factor of motor function (i.e., knee osteoarthritis and patellofemoral pain).

To the best of our knowledge, this is the first systematic review focused on the chronic effects of combining exercise with tDCS in healthy adults. Previous acute studies have demonstrated the positive effects of tDCS on various aspects of motor function, such as strength or endurance [9,10,11]. This acute enhancement in motor function can improve sports performance during competition and may also increase performance during training, influencing exercise-induced adaptations. For example, an acute session of tDCS has been shown to increase not only the total amount of training volume but also to enable a higher concentric movement velocity during a strength-training session [43]. Since training volume and concentric movement velocity during training influence strength training chronic adaptations [44], modulating both variables through tDCS could influence chronic adaptations. However, our systematic review suggests that, in healthy populations, tDCS does not provide additional chronic benefits when combined with exercise in strength, “strength-endurance”, jump height, or balance [23,24,25,26]. Therefore, although tDCS might be considered as an adjuvant method to enhance short-term performance, it may not provide further benefits when used chronically during training sessions. However, it is important to note that only four studies met the inclusion criteria for this review, highlighting the need for further research to establish reliable conclusions.

Stroke patients have lower corticospinal excitability in the affected hemisphere, which usually correlates with chronic poor motor function [45]. Given the potential of tDCS to increase cortical excitability [4,5], this technique could help to reduce symptoms in this population. This hypothesis has been tested by proving the effects of tDCS in combination with walking- [27,28,29,31] and strength-training-based programs [16,17,29,30] on several motor-function tests. When specific tests have been used to determine the effect over a particular domain of motor function, the results do not support the additive effect of tDCS over upper limb dexterity, spasticity, range of motion, or balance [16,17,27]. Although some studies found additive effects from using tDCS during strength training over knee flexion and extension peak torque of the paretic limb [29], no study has found additive effects over the strength of several joint actions nor over multiple-joint lower limb strength tested by the Five Times Sit-to-Stand Test [16,17,30]. When studies have used functional scales that assess multiple domains of motor function, the results are contradictory, with some studies showing positive or no effects of tDCS over tests like the Fugl-Meyer Assessment [16,17,27,28,29,30,31], Wolf Motor-Function Test [16,30], or Performance-Oriented Mobility Assessment [28]. Also, the effects over gait are mixed, with studies showing improved gait speed [28,29] or no additive effects [27,30,31]. Although the high controversy in the results may be related to high heterogeneity in training programs and/or the tests used, overall, the results suggest that tDCS does not enhance the effects of exercise on motor function in this population. These findings align with other systematic reviews and meta-analyses investigating the effects of combining tDCS with other therapies (e.g., virtual reality, physical therapy, or constraint-induced movement therapy) on upper limb spasticity, which report mixed results [19,20,46].

Regarding multiple sclerosis patients, a sense of fatigue is one of the most commonly reported symptoms and is known to interfere with daily activities [47]. Because tDCS has shown promising effects on fatigue reduction [48], its application before or during exercise may increase the overall quality of the rehabilitation session and/or increase exercise-program adherence in patients [34,35]. However, the combination of tDCS with exercise yielded mixed results across different outcome measures. The effects of combining tDCS with exercise appear to enhance balance in patients with multiple sclerosis; however, this outcome has only been measured in one study [33]. Regarding gait, some studies have reported additional benefits of tDCS in combination with exercise, such as improved gait speed or performance on the Timed Up-and-Go test [35,36]. Yet, other studies have not found benefits from including tDCS over gait speed, Timed Up-and-Go Test, Figure-of-Eight Walk Test, or Dynamic Gait Index [32,34]. It is worth noting that the studies reporting additive effects of tDCS during exercise for gait performance did not include walking as a part of the rehabilitation protocol. Therefore, it appears that if any positive effects of tDCS are present, they may be limited when the actual motor task (such as walking) is practised during rehabilitation. These findings are consistent with a systematic review highlighting the positive effects of tDCS on gait speed when applied alone or in combination with cycling, but no significant changes were observed in the Multiple Sclerosis Walking Scale [49].

In Parkinson’s disease, patients experienced a reduction in motor cortical excitability, decreased excitatory signalling from the thalamus to cortical areas, degeneration of dopaminergic neurons, and abnormalities in motor cortical region connectivity [50]. Given the potential of tDCS to modulate these factors, it could help reduce Parkinson’s disease symptoms [50]. However, the effects of tDCS in combination with exercise [gait] have only been tested in one study [37]. The results from this study show no additive effects on bradykinesia, Timed Up-and-Go Test, gait speed, or balance compared to the sham group [37]. Findings from a meta-analysis suggest that tDCS may have positive effects on upper limb motor function, speed, and strength, while more complex tasks may be less affected by tDCS [51]. Another meta-analysis indicated that tDCS interventions can provide benefits for functional locomotion, although the effect sizes were relatively small, and the treatment effects may be enhanced when multiple regions of the motor and prefrontal cortices are targeted [52]. Nevertheless, both meta-analyses synthetize the effects of not only acute and chronic studies but also the tDCS application alone or combined with other protocols (e.g., cognitive training, gait, physical therapy, etc.). From the included studies in these meta-analyses and in our review, it seems that, although tDCS could have a positive effect when applied alone, the additional benefits on motor function are overshadowed by the benefits acquired from gait training without amplifying them.

Age-related changes in motor cortical properties include a decreased corticospinal excitability that may affect motor function [53]. Although tDCS has the potential to modulate this excitability, the only study that met the inclusion criteria did not report a tDCS effect combined with Tai Chi during 12 weeks on gait speed in older adults with mild cognitive impairment [38]. Similarly, another study did not report additional benefits on gait speed when tDCS was applied alone over older adults with mild cognitive and function impairments, although it had an additional effect on balance [54]. However, findings from Rostami et al. [55] indicate the potential benefits of applying tDCS alone for five consecutive days in improving gait, balance, and lower extremity functional performance in healthy older adults. Therefore, although evidence is based only on one study, the possible benefits on gait speed from using tDCS in older people may be limited to a healthy population without relevant cognitive or function impairment.

Following a ligament injury, the individual experiences cortical abnormalities in the somatosensory, motor, and frontal cortex, such as increased motor thresholds and smaller motor-evoked potential amplitudes in the musculature surrounding the injured joint [56]. Considering this, tDCS may be beneficial for people experiencing chronic ankle instability. Only balance benefited from including tDCS plus strength training in one study [40], but not in the other one [39]. Also, no additional benefits were observed in the proprioception, strength, and Side Hop Test in any of the two studies that met the inclusion criteria. Therefore, evidence for the effectiveness of tDCS to increase the magnitude of exercise-induced adaptations is low. Additionally, only two studies were included and both used tDCS during exercise (i.e., online). Further research is needed to determine if priming M1 before exercise can enhance adaptations in this population.

As tDCS could modulate pain [7], combining tDCS with exercise for knee osteoarthritis or patellofemoral pain holds promise for increasing the responsiveness of the brain to the corticomotor benefits of exercise and/or providing additive effects on pain-system function [41,42]. The two studies that investigated the additive effects of combining tDCS with exercise (strength training in both cases) on knee osteoarthritis [41] and patellofemoral pain [42] found additive effects of combining tDCS with exercise on motor function [41] and knee-extensors strength [42]. These findings align with the findings from the Rahimi et al. [57] study, which showed that combining tDCS with a physiotherapy program can improve function in patients with knee osteoarthritis. However, other studies did not find the benefits of applying tDCS alone on knee function [58,59] or gait [59]. Therefore, although evidence is scarce, these results suggest that tDCS could potentially enhance exercise-induced adaptations in those conditions where pain is the limiting factor of motor function, such as knee osteoarthritis or patellofemoral pain.

## 5. Conclusions

The results of the present systematic review suggest that the effectiveness of tDCS to enhance exercise-induced adaptations may depend on the specific combination of treatment modalities and individual patient characteristics. Specifically, chronic use of tDCS in combination with strength training does not seem to enhance motor function in healthy adults. In neurological disorders, the results are contradictory, but, overall, the results suggest that the additive effects may be null if the actual exercise program already includes the functional movements pretending to be improved. However, although evidence is scarce, tDCS may enhance exercise-induced adaptations in those musculoskeletal conditions characterized by pain as a limiting factor of motor function. 

Notwithstanding, these conclusions should be interpreted with caution due to several limitations derived from the included manuscripts. The studies included in this systematic review exhibited considerable variability in terms of tDCS protocols, exercise programs, and outcome measures. Other studies which combined tDCS with exercise were not included in this review due to a lack of specific exercise protocol descriptions (e.g., exercise description, number of repetitions and sets, intensity, etc.), although their results were mixed, as found in this review. Also, many populations include a few or even a single study or had relatively small sample sizes, limiting the statistical power and the generalizability of the findings.

Therefore, the chronic clinical application of this technique needs further investigation to determine if the presumed acute effects on several motor-function capacities have any priming effect over chronic exercise-induced adaptations. This review highlights the need for future investigations with standardized and detailed protocols and larger sample sizes, together with long-term follow-up assessments to enhance the quality of evidence and provide more robust conclusions regarding the chronic effects of tDCS combined with exercise on motor function and performance outcomes.

## Figures and Tables

**Table 1:** Description of methodological quality assessment with PEDRo scale.

Study	PEDro Scale Criteria											Total Score
	1	2	3	4	5	6	7	8	9	10	11	
Hendy and Kidgell [23]	No	Yes	Yes	Yes	Yes	No	Yes	Yes	No	Yes	Yes	8
Jung et al. [24]	Yes	Yes	No	Yes	Yes	No	Yes	Yes	No	Yes	Yes	7
Maeda et al. [25]	No	Yes	No	Yes	Yes	Yes	Yes	Yes	No	Yes	Yes	8
Xiao et al. [26]	Yes	Yes	No	Yes	Yes	Yes	No	Yes	No	Yes	Yes	7
Beaulieu et al. [16]	Yes	Yes	Yes	Yes	Yes	No	Yes	Yes	No	Yes	Yes	8
Madhavan et al. [27]	Yes	Yes	No	Yes	Yes	No	Yes	Yes	Yes	Yes	Yes	8
Manji et al. [28]	Yes	Yes	No	Yes	Yes	No	Yes	Yes	No	Yes	Yes	7
Massaferri et al. [29]	Yes	Yes	No	Yes	Yes	Yes	No	No	No	Yes	Yes	6
Palimeris et al. [17]	Yes	Yes	Yes	No	Yes	Yes	Yes	Yes	No	Yes	No	7
Prathum et al. [30]	Yes	Yes	Yes	Yes	Yes	No	Yes	Yes	No	Yes	No	7
Wong et al. [31]	Yes	Yes	Yes	Yes	Yes	No	Yes	Yes	Yes	Yes	Yes	9
Baroni et al. [32]	Yes	Yes	Yes	Yes	Yes	Yes	Yes	No	Yes	Yes	Yes	9
Marotta et al. [33]	Yes	Yes	Yes	Yes	Yes	No	Yes	Yes	No	Yes	Yes	8
Nguemeni et al. [34]	Yes	Yes	Yes	No	Yes	Yes	No	Yes	Yes	Yes	Yes	8
Pilloni et al. [35]	Yes	Yes	Yes	Yes	Yes	Yes	Yes	No	No	Yes	No	7
Rahimibarghani et al. [36]	Yes	Yes	Yes	Yes	Yes	Yes	Yes	No	Yes	Yes	Yes	9
Costa-Ribeiro et al. [37]	Yes	Yes	Yes	Yes	Yes	Yes	Yes	Yes	No	Yes	No	8
Liao et al. [38]	Yes	Yes	Yes	Yes	Yes	No	Yes	Yes	No	Yes	Yes	8
Bruce et al. [39]	Yes	Yes	Yes	Yes	Yes	No	No	No	No	Yes	Yes	6
Ma et al. [40]	Yes	Yes	Yes	Yes	Yes	Yes	No	Yes	No	Yes	Yes	8
Chang et al. [41]	Yes	Yes	Yes	Yes	Yes	No	No	Yes	Yes	Yes	Yes	8
Rodrigues et al. [42]	Yes	Yes	Yes	No	Yes	No	No	Yes	No	Yes	Yes	6

**Table 2:** Main characteristics of the subjects, protocols and main results related to the motor function or performance in a healthy population.

Study	Sample	tDCS Protocol	Training Protocol	Outcomes	Overall Effect
Hendy and Kidgell [23]	Real tDCS group n = 10 (6 M/4 F) 21.8 ± 0.7 years Sham group n = 10 (5 M/5 F) 25.7 ± 3.1 years	Type: a-tDCS Target electrode: M1 Reference electrode: Supraorbital area Size: 25 cm^2^ Intensity: 2 mA Duration: 20 min Application: Online N° sessions: 9 (3 times per week for 3 weeks)	Dumbbell wrist extension 4 sets of 6–8 repetitions at 70% of 1 RM (3 s concentric and 4 s eccentric)	1 RM dumbbell wrist Extension N.s difference of improvement (% of change) real vs. sham	No
Jung et al. [24]	Real tDCS group n = 27 (12 M/15 F) 40 ± 11.91 years Sham group n = 28 (14 M/14 F) 39.07 ± 12.8 years	Type: a-tDCS Target electrode: M1 Reference electrode: Supraorbital area Diameter: 6 cm Intensity: 2 mA Duration: 20 min Application: Offline (before) N° sessions: 21 (3 times per week for 7 weeks)	Circuit training: 2 sets of as many repetitions as possible in 1 min per exercise (10 s interexercise rest and 30 s interset rest) Exercises: squats, push ups, lunges, band rowing, jump rope, hand walking, sidestep, plank, jump squats, box steps, burpees and in–out jumps.	Isometric elbow flexion strength, isometric knee extension strength, Muscular Fitness Test (sit-ups, push-ups, deep squats and burpees) and Sargent jump N.s for both groups (real and sham) for each pre- and postvalues	No
Maeda et al. [25]	Real tDCS group n = 12 (6 M/6 F) 23.9 ± 1.3 years Sham group n = 12 (6 M/6 F) 23.5 ± 1.4 years	Type: a-tDCS Target electrode: M1 Reference electrode: Ipsilateral upper arm Size: 25 cm^2^ Intensity: 2 mA Duration: 10 min Application: Online. N° sessions: 7 (once every 3 days for 3 weeks)	Eccentric knee flexion and extension on isokinetic machine 3 sets of 10 repetitions with maximum intention at 30°/s	Knee extension and flexion peak torque N.s for intervention x time interaction	No
Xiao et al. [26]	Real tDCS group n = 15 20.5 ± 1.8 years Sham group n = 15 21.3 ± 1.8 years	Type: 4 × 1 ring type HD-tDCS Anodal electrode: Cz Return electrodes: C3, C4, Fz, and Pz Size: 3.14 cm^2^ Intensity: 2 mA Duration: 20 min Application: Online. N° sessions: 12 (3 times per week for 4 weeks)	There was a progression in all exercises across the weeks Foot doming 2 sets of 10–20 repetitions Towel curls 3 sets of 10–20 repetitions with 0–0.5 kg Toe spread and squeeze 2 sets of 10–20 repetitions Balance-board training 2 sets of 20–30 s	Toe flexor strength (*p* < 0.001, 31 ± 19% vs. 9 ± 17%) Significant improvement (% of change) real vs. sham Metatarsophalangeal joint flexor strength and static balance (centre of gravity sway velocity for all conditions and axis) N.s for intervention x time interaction	Not clear

**Table 3:** Main characteristics of the subjects, protocols, and main results related to motor function or performance in a neurological disorder population.

Study	Sample	tDCS Protocol	Training Protocol	Outcomes	Overall Effect
**Stroke**					
Beaulieu et al. [16]	Criteria: Supratentorial stroke > 6 months prior All participants 68.9 ± 10.9 years Real tDCS group n = 7 (5 M/2 F) Sham group n = 7 (5 M/2 F)	Type: a-tDCS Target electrode: Ipsilesional M1 Reference electrode: Contralesional M1 Size: 35 cm^2^ Intensity: 2 mA Duration: 20 min Application: Online N° sessions: 12 (3 times per week for 4 weeks)	Muscles targeted: shoulder flexors, elbow flexors, wrist extensors and grip muscles 1–3 sets of 10–15 repetitions per exercise at 50–80% of 1 RM	Fugl-Meyer Assessment of the Upper Extremity, Wolf Motor-Function Test, Box and Block Test, grip strength and modified Ashworth Scale (shoulder extensors, elbow flexors, wrist flexors, and fingers flexors) N.s in the magnitude of change (real vs. sham)	No
Madhavan et al. [27]	Criteria: Single monohemispheric stroke > 6 months prior Real tDCS group n = 20 (15 M/5 F) 59 ± 9 years Sham group n = 20 (15 M/5 F) 60 ± 9 years	Type: a-tDCS Target electrode: Ipsilesional M1 Reference electrode: Contralesional supraorbital area Size: 12.5 cm^2^ (target) and 24.75 cm^2^ (reference) Intensity: 1 mA Duration: 15 min Application: During ankle motor tracking N° sessions: 12 (3 times per week for 4 weeks)	(1) Ankle motor tracking 14 repetitions of 60 s of skilled visuomotor ankle motor control task (2) High-intensity speed-based treadmill training 40 min walking combining 2 min speed-increasing intervals with variable interset duration rest at 50% maximum speed	10 m Walk Test (comfortable speed), 10 m Walk Test (maximal speed), 6 min Walk Test, Timed Up-and-Go Test, Mini Balance Evaluation Systems Test, and Fugl-Meyer Assessment of the Lower Extremity N.s for intervention x time interaction	No
Manji et al. [28]	Criteria: Poststroke hemiparetic patients with new-onset supratentorial lesion Real tDCS group n = 15 (10 M/5 F) 62.2 ± 10.1 years Sham group n = 15 (11 M/4 F) 63.7 ± 11.0 years	Type: a-tDCS Target electrode: Supplementary motor area Reference electrode: Inion Size: 25 cm^2^ Intensity: 1 mA Duration: 20 min Application: Online N° sessions: 7 (Once a day for 1 week)	Body weight-supported treadmill training 20 min walking with 20% body weight support at 80–90% maximum speed	10 m Walk Test (*p* < 0.001 group A and *p* = 0.001 group B) and Timed Up-and-Go Test (*p* < 0.001 groups A and B) Significant improvement for real period of intervention and n.s for a sham period of intervention between pre- and post-testing Fugl-Meyer Assessment of the Lower Extremity, Trunk Impairment Scale, and Performance-Oriented Mobility Assessment N.s for intervention x time interaction	Not clear
Massaferri et al. [29]	Criteria: Hemiparesis within at least 6 months of the stroke occurrence Real tDCS group n = 10 (6 M/4 F) 53.8 ± 12.2 years Sham group n = 8 (6 M/2 F) 58.1 ± 6.5 years	Type: a-tDCS Target electrode: Ipsilesional M1 Reference electrode: Contralesional M1 Size: 35 cm^2^ Intensity: 2 mA Duration: 20 min Application: Offline (before) N° sessions: 24 (Twice a week for 12 weeks)	(1) 20 min treadmill walking at GET (2) Manual skills mimicking daily tasks (3) 2 sets of 10–15 repetitions at 75% 15 RM of the following exercises: step up, squat, leg press, seated row, knee extension and flexion, chest press, shoulder press, biceps curl, and hip abduction (4) Stretching	Fugl-Meyer Assessment (Total score), Fugl-Meyer Assessment of the Lower Extremity, 10 m Walk Test, W_max_ (treadmill), W-GET (treadmill), knee flexion and extension peak torque (paretic limb), and extension bilateral deficit *p* < 0.05 for the real group and n.s for the sham group between pre- and post testing VO_2max_, VO_2-GET_, knee flexion and extension peak torque (nonparetic limb) and flexion bilateral deficit N.s for both groups (real and sham) between pre- and post-testing	Not clear
Palimeris et al. [17]	Criteria: Single unilateral stroke > 6 months prior All groups 65.3 ± 12.0 years Real tDCS group n = 48 Sham group n = 42	Type: a-tDCS Target electrode: Ipsilesional M1 Reference electrode: Contralesional supraorbital area Size: 35 cm^2^ Intensity: 2 mA Duration: 20 min Application: Online N° sessions: 12 (3 times per week for 4 weeks)	3 sets of 10 repetitions at RPE 12–16 (6–20 Scale) of the following exercises: wrist extension, biceps curl, shoulder flexion, and hand gripping Intensity varied depending on MEP amplitude (35–85% 1 RM)	Fugl-Meyer Stroke Assessment Scale, grip strength, Box and Block Test, and active and passive range of motion (shoulder flexion, elbow flexion, and wrist extension) N.s for intervention x time interaction	No
Prathum et al. [30]	Criteria: First ischemic stroke onset between 6 months and 5 years ago Real tDCS group n = 12 (8 M/4 F) 58.67 ± 3.70 Sham group n = 12 (8 M/4 F) 56.83 ± 3.58 years	Type: a-tDCS Target electrode: Ipsilesional M1 Reference electrode: Contralesional M1 Size: 35 cm^2^ Intensity: 2 mA Duration: 20 min Application: Offline (before) N° sessions: 12 (3 times per week for 4 weeks)	(1) 2 min stretching per muscle group: elbow flexors, wrist flexors, shoulder flexors, hip extensors, knee flexors, and ankle plantar flexors (2) 3 sets of 10 repetitions of the following movements: elbow extension, shoulder flexion, forearm pronation, forearm supination, sit to stand, step forward, and step sideward (3) 50 repetitions per direction of reach-to-grasp exercise in 3 different directions	Fugl-Meyer Stroke Assessment of the Upper Extremity (*p =* 0.029), Fugl-Meyer Stroke Assessment of the Lower Extremity (*p* = 0.024), Fugl-Meyer Stroke Assessment Scale Total Score (*p* = 0.009 and Wolf Motor-Function Test in functional ability scale (*p* = 0.043) Significant intervention x time interaction in favour of real group Wolf Motor-Function Test in performance time, Timed Up-and-Go Test, 6 m Walk Test, Five Times Sit-to-Stand Test, grip strength and muscle strength of wrist extensor, elbow extensor, ankle dorsiflexor, knee extensor, hip flexor, and hip extensor N.s for intervention x time interaction	Not clear
Wong et al. [31]	Criteria: 6 months after first-ever stroke with unilateral motor deficits Bilateral a-tDCS group n = 14 (10 M/4 F) 55.43 ± 5.9 years Cathodal tDCS group n = 14 (11 M/3 F) 60.64 ± 11.3 years Sham group n = 14 (11 M/3 F) 64.05 ± 9.4 years	Type: bilateral a-tDCS Target electrode: Ipsilesional M1 Reference electrode: Contralesional M1 Type: c-tDCS Target electrode: Contralesional M1 Reference electrode: Ipsilesional supraorbital area Size: 35 cm^2^ Intensity: 2 mA Duration: 20 min Application: Offline (before) N° sessions: 12 (3 times per week for 4 weeks)	Treadmill walking 30 min increasing 0.2 km/h per 5 min from comfortable speed to RPE 13 (6–20 Scale)	Test: comfortable walk speed (outcomes: speed, cadence, and unaffected leg step time) Significantly increased cadence (*p* = 0.026) and decreased unaffected leg step time (*p* = 0.003) in the cathodal group vs. sham group. Significantly increased speed (*p* = 0.005) and cadence (*p* = 0.017) in the cathodal group vs. bilateral group. n.s for the rest of comparisons Test: motor dual-task walk speed (all parameters) and comfortable walk speed (outcomes: both legs’ step length and step time affected leg) N.s for intervention x time interaction Fugl-Meyer Assessment of Lower Extremity Only the cathodal group significantly improved (*p* = 0.002) from pre- to post-testing	Not clear
**Multiple sclerosis**					
Baroni et al. [32]	Criteria: Multiple sclerosis patients without relapses 3 months prior Real tDCS group n = 8 (4 M/4 F) 55.25 ± 15.15 years Sham group n = 8 (4 M/4 F) 52.13 ± 11.31 years	Type: a-tDCS Target electrode: 3 cm lateral to the inion (right cortex) Reference electrode: Right buccinators muscle Size: 35 cm^2^ Intensity: 2 mA Duration: 15 min Application: Online N° sessions: 10 (5 times per week for 2 weeks)	(1) Circuit training 2 sets of 3 min work per exercise (2 min interexercise rest and 10 min interset rest) Exercises: step, slalom exercise, tandem walking, one-leg balance, walk between obstacles, and walk with long steps (2) 30 min treadmill walking at a self-selected speed between 0.9 and 2.9 km/h	Timed Up-and-Go Test, Figure-of-Eight Walk Test and Dynamic Gait Index N.s for intervention x time interaction	No
Marotta et al. [33]	Criteria: Relapsing–remitting multiple sclerosis patients Real tDCS group n = 9 (3 M/6 F) 43.22 ± 10.46 years Sham group n = 8 (2 M/6 F) 39.75 ± 8.39 years	Type: a-tDCS Target electrode: Most affected M1 Reference electrode: Contralateral supraorbital area Size: 25 cm^2^ Intensity: 2 mA Duration: 20 min Application: Offline (before) N° sessions: 10 (5 times per week for 2 weeks)	(1) 30 min of proprioceptive, static, and dynamic balance exercises with a computerized board (2) Exercises: knee flexion and extension, plantarflexion, trunk flexion, and trunk extension 5–10 reps at 60% 1 RM. Number of sets not specified (3) 3 min treadmill walking	6 min Walk Test distance (*p* = 0.006 real and *p* = 0.009 sham) and Timed Up-and-Go Test (*p* = 0.031 real and *p* = 0.043 sham) Significant differences between pre- and post-testing for both groups (real and sham) Berg Balance Scale *p* = 0.023 for the real group and n.s for the sham group between pre- and post-testing 6 min Walk Test (velocity and distance of gait cycle) N.s for both groups (real and sham) between pre- and post-testing	Not clear
Nguemeni et al. [34]	Criteria: Multiple sclerosis patients with a stable condition 3 months prior Real tDCS group n = 12 (7 M/5 F) 49.83 ± 10.46 years Sham group n = 10 (3 M/7 F) 46.90 ± 9.00 years	Type: a-tDCS Target electrode: 3 cm lateral to the inion (hemisphere ipsilateral to the fast leg) Reference electrode: Ipsilateral buccinators muscle Size: 25 cm^2^ Intensity: 2 mA Duration: 15 min Application: Offline (before) N° sessions: 6 (3 times per week for 2 weeks)	Split Belt Treadmill Program (1) 2 min walking at a slow speed and 2 min at a fast speed (both belts same speed) (2) 20 min asymmetrical walk with 1:2 slow–fast ratio (1 min rest after 10 and 15 min) (3) 50 m walk on ground	Functional Gait Assessment, Timed Up-and-Go Test, 50 m Walk Test, and 2 min Walk Test N.s for intervention x time interaction	No
Pilloni et al. [35]	Criteria: Relapsing–remitting or secondary progressive multiple sclerosis patients Real tDCS group n = 9 (3 M/6 F) 52.1 ± 12.8 years Sham group n = 6 (1 M/5 F) 53.5 ± 9.8 years	Type: a-tDCS Target electrode: M1 Reference electrode: Supraorbital area Size: 25 cm^2^ Intensity: 2.5 mA Duration: 20 min Application: Online N° sessions: 10 (5 times per week for 2 weeks)	20 min at 60–80% HR_max_ in a recumbent combination arm/leg elliptical ergometer	10 m Walk Test (gait speed, stride length, gait cycle duration and cadence) and 2 min Walk Test (all parameters) *p* < 0.05 for the real group (all parameters) and n.s for the sham group between pre- and post-testing 10 m Walk Test (stance phase and double support phase) N.s for intervention x time interaction	Not clear
Rahimibarghani et al. [36]	Criteria: Multiple sclerosis patients Real tDCS group n = 21 (8 M/13 F) 40.0 ± 7.1 years Sham group n = 18 (7 M/11 F) 39.8 ± 6.6 years	Type: a-tDCS Target electrode: Dominant M1 Reference electrode: Opposite shoulder Size: Target electrode 16 cm^2^ and reference electrode 36 cm^2^ Intensity: 1.5 mA Duration: 20 min Application: Offline N° sessions: 12 (2 times per week for 6 weeks)	10 min of cycling at 30 W	Timed Up-and-Go Test (*p* = 0.02, 23.2% vs. 8.3%) and 2-Minute Walk Test (*p* = 0.02, −16.9% vs. −8.5%) Significant improvement (% of change) real vs. sham 5-Meter Walk Test (−13.6% vs. −6.7%) N.s difference of improvement (% of change) real vs. sham	Not clear
**Parkinson**					
Costa-Ribeiro et al. [37]	Criteria: Parkinson patients (40–80 years) in 1–3 Hoehn and Yahr stage Real tDCS group n = 11 (8 M/3 F) 61.1 ± 9.1 years Sham group n = 11 (7 M/4 F) 62.0 ± 16.7 years	Type: a-tDCS Target electrode: 2 cm anterior to the vertex Reference electrode: Supraorbital area contralateral to the more affected side Size: 35 cm^2^ Intensity: 2 mA Duration: 13 min Application: Offline (before) N° sessions: 10 (≈ 3 times per week for 4 weeks)	Gait training with visual cues 3 sets of 8 min (2 min interset rest) Stripes were set at 40% of individuals’ height and increased by 20% every 3 sessions	10 m Walk Test (all parameters), Timed Up-and-Go Test, bradykinesia subscores of Unified Parkinson’s Disease Rating Scale, an upper limb motor-task time, and Berg Balance Scale N.s for intervention x time interaction	No
**Old people with mild cognitive impairment**					
Liao et al. [38]	Criteria: Over 65 and with mild cognitive impairment Real tDCS group n = 10 (2 M/8 F) 72.6 ± 4.1 years Sham group n = 10 (5 M/5 F) 73.1 ± 4.6 years	Type: a-tDCS Target electrode: M1 Reference electrode: Supraorbital area Size: 35 cm^2^ Intensity: 2 mA Duration: 20 min Application: Offline (before) N° sessions: 36 (3 times per week for 12 weeks)	20 min of Tai Chi	Walk at the subject’s preferred gait speed (all parameters) and motor dual-task walking (all parameters) N.s for intervention x time interaction	No

GET: gas-exchange threshold; HR_max_: maximum heart rate; MEP: motor evoked potential; VO_2-GET_: maximum oxygen consumption at gas-exchange threshold; VO_2max_: maximum oxygen consumption; W_max_: external work performed at maximum oxygen consumption; W-GET: external work performed at gas-exchange threshold.

**Table 4:** Main characteristics of the subjects, protocols, and main results related to motor function or performance in the musculoskeletal disorder population.

Study	Sample	tDCS Protocol	Training Protocol	Outcomes	Overall Effect
**Chronic ankle instability**					
Bruce et al. [39]	Criteria: Ankle sprain more than 1 year prior and recurrent sensations of rolling or giving way Real tDCS group n = 13 (3 M/10 F) 22.2 ± 2.8 years Sham group n = 13 (6 M/7 F) 22.5 ± 3.2 years	Type: a-tDCS Target electrode: M1 Reference electrode: Supraorbital area Size: 15 cm^2^ Intensity: 1.5 mA Duration: 18 min Application: Online N° sessions: 10 (5 times per 2 weeks for 4 weeks)	Eccentric ankle eversion on an isokinetic dynamometer 4 sets of 10 repetitions at 60% of their maximal eccentric torque and with no resistance on the concentric phase	Dynamic Postural Stability Index No effect (significant intervention x time interaction without post hoc significance) Side hop Test and concentric and eccentric inversion and eversion strength at 30°/s and 90°/s N.s for intervention x time interaction	No
Ma et al. [40]	Criteria: 2 or more sprains 3 months prior Real tDCS group n = 14 (7 M/7 F) 21.14 ± 2.82 years Sham group n = 14 (6 M/8 F) 20.29 ± 1.49 years	Type: 4 × 1 ring type HD-tDCS Anodal electrode: Cz Return electrodes: C3, C4, Fz, and Pz Size: 5 mm radius Intensity: 2 mA Duration: 20 min Application: Online N° sessions: 12 (3 times per week for 4 weeks)	Short foot exercise (i.e., to shorten the foot in the anterior–posterior direction without flexing the toes) 4 sets of 12 repetitions The intensity was increased from sitting to one-leg standing	Active Movement Extent Discrimination Apparatus, Joint Position Reproduction (10° inversion and 15° eversion), and Sensory Organization test (SOT1, SOT2, SOT3, and SOT6) N.s for intervention x time interaction The Joint Position Reproduction (15° inversion) No effect (significant intervention x time interaction without post hoc significance) Y-Balance Test (*p* = 0.004) and SOT4 and SOT5 Sensory Organization Test (*p <* 0.05 and *p* < 0.05) Significant improvement for the real group and n.s for the sham group between pre- and post-testing	Not clear
**Knee osteoarthritis**					
Chang et al. [41]	Criteria: People with knee osteoarthritis Real tDCS group n = 15 (4 M/11 F) 59.8 ± 9.1 years Sham group n = 15 (6 M/9 F) 64.1 ± 11.1 years	Type: a-tDCS Target electrode: Contralateral M1 to the side of the worst knee Reference electrode: Contralateral supraorbital area Size: 35 cm^2^ Intensity: 1 mA Duration: 20 min Application: Offline (before) N° sessions: 16 (Twice a week for 8 weeks)	Exercises: Knee extension, hip abduction, squat, isometric band leg curl, and step up 3 sets of 10 repetitions per exercise The intensity was adjusted with ankle weights, bands and body weight	Western Ontario and McMaster Universities Osteoarthritis Index Physical Function Subscale *p* < 0.05 for the real group and n.s for the sham group between pre- and post-testing	Yes
**Patellofemoral pain**					
Rodrigues et al. [42]	Criteria: Women between 18–30 with pain in the patellofemoral joint Real tDCS group n = 14 F 21.7 ± 63.4 years Sham group n = 14 F age 24.16 ± 3.9 years	Type: a-tDCS Target electrode: Cz Reference electrode: Orbitofrontal cortex contralaterally to the dominant leg Size: 35 cm^2^ Intensity: 2 mA Duration: 20 min Application: Offline (before) N° sessions: 12 (2–3 times per week)	Knee extension 3 sets of 12 repetitions at 60% 1 RM (2 s concentric and 2 s eccentric)	10 RM Knee Extension Significant intervention x time interaction in favour of real, *p* < 0.05	Yes

## Data Availability

No new data were created or analyzed in this study. Data sharing is not applicable to this article.
